# WNTA5-mediated miR-374a-5p regulates vascular smooth muscle cell phenotype transformation and M1 macrophage polarization impacting intracranial aneurysm progression

**DOI:** 10.1038/s41598-024-51243-z

**Published:** 2024-01-04

**Authors:** Zengshi Li, Junqiang Huang, Lijian Yang, Xi Li, Wei Li

**Affiliations:** Department of Neurosurgery, The Affiliated Changsha Hospital of Xiangya School of Medicine, Central South University, Changsha, China

**Keywords:** Cell biology, Oncology

## Abstract

miR-374a-5p expression and localization in intracranial aneurysm (IA) tissues were detected, and its correlation with vascular smooth muscle cells (VSMCs) and macrophage markers was analyzed. Using platelet-derived growth factor-BB (PDGF-BB) induced VSMC model, elastase-induced IA rat model. Subsequently, miR-374a-5p was knocked down or overexpressed. We investigated the effects of miR-374a-5p on phenotypic conversion, and in vivo experiments were also carried out to verify the findings. The targeted relationship between miR-374a-5p and WNTA5 was analyzed. The effect of WNT5A inhibition on VSMC phenotypic transformation and THP-1-derived macrophage polarization was explored. Clinical studies have shown that miR-374a-5p was upregulated in IA patients. miR-374a-5p was negatively correlated with SM22α, α-SMA, CD206, and positively correlated with CD86. In vitro experiments showed that knocking down miR-374a-5p reversed the promotion of SM22α and α-SMA expression by PDGF-BB, while overexpression of miR-374a-5p had the opposite effect. In addition, knocking down miR-374a-5p also reversed the decrease in Calponin, TIMP3, TIMP4, and IL-10 levels caused by PDGF-BB, and further reduced the levels of MMP1, MMP3, MMP9, IL-1β, IL-6, and TNF-α. These findings were further validated in vivo. In IA rats, there were notable increases in both systolic and diastolic blood pressure, along with an elevated M1/M2 ratio and the occurrence of vascular lesions. However, these symptoms were improved after knocking down miR-374a-5p. Furthermore, miR-374a-5p could target the WNT signals (WNT2B, WNT3, and WNT5A). miR-374a-5p regulated the VSMC phenotypic conversion and M1 macrophage polarization by targeting WNT5A, thereby impacting the progression of IA.

## Introduction

Intracranial aneurysm (IA) represents the primary trigger of subarachnoid hemorrhage, a condition linked to high morbidity and mortality^1^. The exact mechanisms responsible for the development and rupture of IA are still not entirely comprehended. However, it is widely believed that endothelial dysfunction, the phenotypic transformation of vascular smooth muscle cells (VSMCs), secretion of matrix metalloproteinases, and activation of innate immune cells contribute to the pathological changes observed in the vascular system^2^. Multiple studies have shown that VSMC phenotypic transformation serves as essential for the formation and progression of IA, and modulation of VSMC phenotypic transformation can attenuate the formation of IA^3,4^. In addition, macrophage polarization is implicated in the formation and rupture of IA^2^. It has been demonstrated that an M1-polarized macrophage increases the risk of IA rupture, while polarization towards an M2 phenotype can prevent IA rupture^5,6^.

MicroRNAs (miRNAs), as a class of endogenous non-coding RNAs, play a crucial role in the regulation of gene expression. They have been identified as potentially valuable biomarkers for disease diagnosis, prognosis, and therapeutic interventions^7,8^. Currently, there is evidence suggesting biological connections between miRNAs and IA. For example, miR-34a modulates VSMC phenotypic transformation in IA by targeting CXCR3 and MMP-2^9^. miR-21 regulates the formation and rupture of IA in response to an inflammatory response mediated via the JNK signaling pathway^10^. miR-139-5p inhibits proliferation and angiogenesis in human brain microvascular endothelial cells by suppressing FGB, thus preventing IA formation^11^. Recent research has indicated a significant upregulation of miR-374a-5p in IA patients^12^. Despite this, the precise role and mechanism by which miR-374a-5p contributes to IA are not yet thoroughly understood and necessitate further investigation for clarification.

WNT signaling pathway, including both the WNT/β-Catenin dependent and β-catenin independent pathways, exerts a pivotal influence on various cellular processes, such as inflammation regulation, cell fate determination, proliferation, differentiation, cell polarity, and migration^13^. Numerous reports demonstrated a critical role of the canonical WNT signaling pathway in cardiovascular disease, specifically in the biology of VSMCs^14^. A previous study found that the proliferation rate of VSMCs overexpressing WNT5A was significantly accelerated, while using si-WNT5A to knock down WNT5A reversed this proliferative effect^15^. Ackers et al. found that WNT5A and WNT3A differentially regulate VSMC migration and proliferation, and competitively regulate VSMC phenotypic transformation^16^. Carthy et al. discovered that WNT3A increased the expression of contractile proteins, calponin, and alpha-smooth muscle actin (α-SMA), promoting the contractile and secretory phenotype of VSMCs^17^. Moreover, elevated WNT5B expression is linked to vascular remodeling and promotes VSMC phenotypic transformation in CTEPH by regulating mitochondrial dynamics^18^. Previous studies have also found that WNT signaling activation promotes M2 macrophage polarization^19,20^. Nevertheless, the significance of WNT signaling in the pathophysiology of IA remains poorly understood.

Platelet-derived growth factor-BB (PDGF-BB) is an effective inducer of VSMC phenotypic transformation^21^. The expression of PDGF-BB in the vasculature is upregulated during arterial remodeling in the context of atherosclerosis^22^. This study first identified the expression of miR-139-5p in IA and investigated the role of miR-139-5p in PDGF-BB-induced VSMC phenotypic transformation and THP-1-derived macrophage polarization. The involvement of WNT5A in PDGF-BB-induced VSMC phenotypic transformation and THP-1-derived macrophage polarization was also examined using the WNT5A antagonist Box5. The ultimate objective is to develop novel treatment strategies and identify potential targets for managing IA.

## Material and methods

### Collection of clinical specimens

IA patients (n = 2) were recruited, and age-matched healthy individuals were included as controls (n = 4). Tissue samples were collected from all subjects. This study was approved by the Affiliated Changsha Hospital of Xiangya School of Medicine, Central South University, and followed the 1964 Declaration of Helsinki. Written informed consent has been obtained from all participants.

### Animals and treatment

Male Sprague-Dawley rats aged 8 weeks and bred under SPF conditions were selected from Hunan SJA Experimental Animal Co., Ltd, and divided randomly into four groups (n = 10 per group): Sham, IA, IA+ inhibitor NC, and IA+ miR-374a-5p inhibitor groups. Rats in the Sham group underwent sham surgery and were injected stereotactically with 1 μL PBS once per day. In the IA group, rats received stereotactic injection of 1 μL of a mixture of PBS and Lipofectamine 2000 once daily. In the IA+ inhibitor NC group, rats received a stereotactic injection of 1 μL of a mixture of inhibitor NC (4.5 μM) and Lipofectamine 2000 once a day. In the IA+ miR-374a-5p inhibitor group, rats received a stereotactic injection of 100 μL of a mixture of miR-374a-5p inhibitor (4.5 μM) and Lipofectamine 2000 once a day. The IA model was induced by elastase (E1250, Sigma-Aldrich) in the other groups. In brief, the rats were anesthetized with 3% isoflurane and positioned supinely, following which the right carotid artery was ligated with a 4–0 nylon suture. One week later, a small single hole was drilled, and 10 μL of elastase was injected into the right basal cistern according to the stereotactic coordinates^3,23^. A high salt diet (8% NaCl and 0.12% β-aminopropionitrile^23^) was provided to induce hypertension in rats for 30 days. Measurements for baseline systolic blood pressure (SBP) and diastolic blood pressure (DBP) were taken at different time points: weeks 0, 1, 2, 3, and 4, after IA induction. After 30 days, rats were euthanized with CO_2_ overdose. Subsequently, a subset of rats from each group (n = 3 per group) underwent perfusion fixation using PBS and 4% paraformaldehyde (PFA), followed by treatment with 2% India ink^4^. The perfused and treated samples were then used for subsequent tissue pathology analysis. The animal study was conducted with the approval of the ethics review committee of The Affiliated Changsha Hospital of Xiangya School of Medicine, Central South University (2022-7). All methods are implemented in accordance with ARRIVE guidelines and regulations.

### Cell culture

Human brain VSMCs (CP-H116, Procell) were cultured for 24 h in a humidified incubator at 37 ℃, 5% CO_2_ in Ham’s F-12 K medium (iCell-0007, ICell). The Ham’s F-12 K basal medium was composed of 0.05 mg/mL ascorbic acid, 0.01 mg/mL insulin, 0.01 mg/mL transferrin, 10 ng/mL sodium selenite, 20% fetal bovine serum, and 10 mM HEPES and TES^16^. Afterward, the medium was substituted with serum-free F-12 K supplemented with 10 ng/mL of PDGF-BB (100-14B, Peprotech) and incubated at 37 ℃ for 24 h to induce a phenotype transformation^3^. VSMCs were then cultured in F-12 K with 10% FBS for experimental analysis. For the experiment, the cells were allocated to six groups, each with a different treatment: (1) Control group (Culture normally without any additional treatment), (2) Model group (Induction with 10 ng/mL PDGF-BB), (3) inhibitor NC group (Transfection with inhibitor negative control (NC) for 48 h, then induction with 10 ng/mL PDGF-BB), (4) miR-374a-5p inhibitor group (Transfection with miR-374a-5p inhibitor for 48 h, then induction with 10 ng/mL PDGF-BB), (5) mimics NC group (Transfection with mimics NC for 48 h, then induction with 10 ng/mL PDGF-BB), (6) mimics NC group (Transfection with miR-374a-5p mimics for 48 h, then induction with 10 ng/mL PDGF-BB), (7) miR-374a-5p inhibitor + Vehicle group (Transfection with miR-374a-5p inhibitor for 48 h, intervention with 100 μM/L physiologic saline for 2 h, and finally induction with 10 ng/mL PDGF-BB), (8) miR-374a-5p inhibitor + Box5 group (Transfection with miR-374a-5p inhibitor for 48 h, intervention with 100 μM/L Box5 for 2 h^24^, and finally induction with 10 ng/mL PDGF-BB).

The THP-1 cell line, a human monocytic leukemia cell line, was cultured at 37 ℃ and 5% CO_2_. The RPMI-1640 medium used in the culture process was supplemented with 10% FBS. To trigger the differentiation of monocytes into macrophages, the THP-1 cells were exposed to 50 ng/mL phorbol-12-myristate-13-acetate (PMA) for 24 h^16^. Subsequently, the VSMCs treated as described above were co-cultured with THP-1 cells in a 2:1 ratio. The co-cultured cells were then divided into four groups: THP-1 + inhibitor NC group, THP-1 + miR-374a-5p inhibitor group, THP-1 + miR-374a-5p inhibitor + Vehicle group, and THP-1 + miR-374a-5p inhibitor + Box5 group.

### Cell transfection

Using Lipofectamine 2000 (11668019, Invitrogen), according to the manufacturer’s instructions, miR-374a-5p mimics (HG-hm374a-5p-mi, HonorGene), miR-374 (a)-5p inhibitor (Human: HG-hm374a-5p-in, Rat: HG-ro374-5p-in, Honor Gene) were transfected into cells to achieve overexpression and knockdown of miR-374a-5p. The sequence for the miR-374a-5p mimics is UUAUAAUACAACCUGAUAAGUG. The sequence for the miR-374 (a)-5p inhibitor differs between species: CACUUAUCAGGUUGUAUUAUAUAA for humans and CACUUAGCAGGUUGUAUUAUAU for rats. Mimics NC and inhibitor NC were utilized as controls. After 6 h of transfection, the normal culture medium was replaced for further cultivation, and relevant analysis was conducted 48 h later.

### Flow cytometry

For the detection of M1 and M2 macrophage infiltration, flow cytometry was performed. The cells were first rinsed with 1 mL of PBS after 1 × 10^5^ cells (100 µL) were added in a 1.5 mL EP tube, followed by centrifugation at 1500 rpm for 5 min to obtain the sediment. After resuspending the cells in 100 µL of culture medium, CD86+ and CD206+ antibodies were added and incubated in the dark under room temperature for 30 min. The cell pellet, after washing with PBS and undergoing centrifugation, was ultimately suspended in 150 µL of basal medium for flow cytometry analysis.

### Quantitative real-time PCR (qRT-PCR)

In this study, the TRIzol total RNA extraction kit (15596026, Thermo) was used to extract total RNA from the cells and tissues, followed by reverse transcription of the total mRNA into cDNA with the cDNA reverse transcription kit (CW2569, CWBIO). The primers for the target gene were designed using the Primer5 software, based on the sequence obtained from NCBI and synthesized by Beijing Tsingke. For relative gene expression analysis, the Ultra SYBR Mixture (CW2601, CWBIO) was used on a fluorescence quantitative PCR machine (QuantStudio1, Thermo). H-GAPDH, H-U6 or R-5S were utilized as internal reference genes to obtain the relative levels of the target gene, calculated using the 2^−ΔΔCt^ method with the help of their respective standard curves. Table 1 shows the primer sequences employed during the study.

**Table 1:** Primer sequences.

Gene	Primer sequences	Length
R-miR-374-5p	FAUAUAAUACAACCUGCUAAGUG	100 bp
	RGCTGTCAACGATACGCTACGTAA	
R-5S	FGCCTACAGCCATACCACCCGGAA	116 bp
	RCCTACAGCACCCGGTATCCCA	
H-miR-374a-5p	FTTATAATACAACCTGATAAGTG	71 bp
	RGCTGTCAACGATACGCTACGTAA	
H-U6	FCTCGCTTCGGCAGCACA	94 bp
	RAACGCTTCACGAATTTGCGT	
H-CD86	FCTGTCCACCCCATCAACAAGTCT	198 bp
	RAGCCTCCTTCCATTCATCCCAT	
H-CD206	FTATGCCAGACACGATCCGACCC	135 bp
	RAGTATGTCTCCGCTTCATGCCAT	
H-α-SMA	FCTATGAGGGCTATGCCTTGCC	122 bp
	RGCTCAGCAGTAGTAACGAAGGA	
H-SM22α	FCCTCTGACACATGCGGCTT	114 bp
	RAGTCATTCCAGGTCGGCATC	
H-WNT2B	FAAAAGGGGCCAGGAGGATTC	138 bp
	RGCTGGCTCTTGCTTGCTTAC	
H-WNT3	FACTTTTGTGAGCCCAACCCA	130 bp
	RTTCTCCGTCCTCGTGTTGTG	
H-WNT5A	FTCCTCTCGCCCATGGAATTA	106 bp
	RCATTGCACTTCCAGCCATCC	
H-GAPDH	FACAGCCTCAAGATCATCAGC	104 bp
	RGGTCATGAGTCCTTCCACGAT	

### Western blot (WB)

To extract proteins from tissues and cells, RIPA lysis buffer was utilized, followed by protein quantification using the BCA protein assay kit. Electrophoresis-separated protein lysates were transferred to nitrocellulose membranes, which were then blocked using 5% skim milk at room temperature for 90 min. Primary antibodies such as Calponin, TIMP3, TIMP4, SM22α, α-SMA, MMP1, MMP3, MMP9, WNT2B, WNT3, and WNT5A were used with GAPDA serving as the internal control. The membranes were left overnight in primary antibodies at 4 ℃, followed by washing with PBST. Secondary antibodies (HRP goat anti-mouse IgG and HRP goat anti-rabbit IgG) were diluted and left to incubate on the membrane for 90 min at room temperature. Following three PBST washes, the membrane was visualized and analyzed for grayscale using Quantity One software (Fig. S1). Antibody information is shown in Table 2.

**Table 2:** Antibodies used in the study are shown.

Indicator	Dilution	Origin	Manufacturer
Calponin	1:5000	Rabbit	24855-1-AP, Proteintech
TIMP3	1:1000	Rabbit	10858-1-AP, Proteintech
TIMP4	1:1000	Rabbit	12326-1-AP, Proteintech
SM22α	1:5000	Rabbit	ab155272, Abcam
α-SMA	1 μg/mL	Rabbit	ab5694, Abcam
MMP1	1:1000	Rabbit	26585-1-AP, Proteintech
MMP3	1:1000	Rabbit	ab53015, Abcam
MMP9	1:1000	Rabbit	27306-1-AP, Proteintech
WNT2B	1:3000	Rabbit	ab178418, Abcam
WNT3	1 µg/mL	Rabbit	ab32249, Abcam
WNT5A	1:3000	Rabbit	ab235966, Abcam
GAPDH	1:5000	Rabbit	10494-1-AP, Proteintech
HRP goat anti-mouse IgG	1:5000	Mouse	SA00001-1, Proteintech
HRP goat anti-rabbit IgG	1:6000	Rabbit	SA00001-2, Proteintech

### Haematoxylin–eosin (HE) staining

Fixed tissue samples were embedded in paraffin and sectioned to 6 μm thickness. After treatment with xylene and dehydration using different concentrations of ethanol (100%, 95%, 85%, and 75%) for 5 min at each level, the sections were subjected to hematoxylin staining and eosin staining for 1 min, respectively. Gradual alcohol dehydration (95–100%) was then performed for 5 min at each level. Finally, the sections were washed with xylene twice for 10 min each and sealed with neutral gum for microscopic observation.

### Immunofluorescence (IF) assay

To assess SM22α and α-SMA expression in cells, IF was employed. Slides were fixed with 4% paraformaldehyde for 30 min, blocked with 0.3% Triton X-100 at 37 ℃ for 30 min, and incubated in a 5% BSA blocking solution for 1 h. They were then incubated overnight at 4 ℃ with primary antibodies against SM22α (ab155272, 1:50, Abcam) and α-SMA (BM0002, 1:50, BOSTER). Subsequently, slides were incubated with secondary antibodies (Alexa Fluor 488-conjugated Goat Anti-Rabbit IgG(H + L) (AWS0005b) and Alexa Fluor 594-conjugated Goat Anti-Mouse IgG(H + L) (AWS0004b)) from Abiowell, diluted 1:200, for 90 min at 37 ℃. Finally, slides were sealed with glycerol and observed using a fluorescence microscope (BA410T, Motic) in a dark environment.

### Fluorescence in situ hybridization (FISH) and IF (FISH-IF)

As directed by the manufacturer, RNA FISH was carried out using a FISH kit (Ribo Bio). For IF staining, the dewaxed and dehydrated paraffin sections were heated in 0.01 M citrate buffer (pH 6.0) for 20 min. After reacting with the blocking agent, the sections were incubated with SM22α (ab283654, 1:50, Abcam) and CD68 (ab170902, 1:50, Abcam) antibodies at 37 °C overnight. Then, they were incubated with secondary antibody CoraLite488-conjugated Affinipure Goat Anti-Rabbit IgG (H + L) (SA00013-2, Proteintech) at 37 °C for 90 min. The co-localization of miR-374A-5p with SM22α and CD68 was detected by fluorescence microscope.

### Enzyme-linked immunosorbent assay (ELISA)

Levels of TNF-α, IL-10, IL-1β, IL-6, and WNT5A concentrations in cell supernatants were examined using TNF-α kit (CSB-E04740h), IL-1β kit (CSB-E08053h), IL-6 kit (CSB-E04638h), and WNT5A kit (CSB-EL026138HU) bought from Wuhan CUSABIO, and IL-10 kit (KE00170) obtained from Proteintech, following the manufacturer’s protocols. Absorbance measurements were captured at a wavelength of 550 nm utilizing a microplate reader. Similarly, using corresponding kits from CUSABIO (Wuhan, China): TNF-α kit (CSB-E11987r), IL-1β kit (CSB-E08055r), IL-6 kit (CSB-E04640r), and IL-10 kit (CSB-E04595r), the levels of TNF-α, IL-10, IL-1β, and IL-6 in rat serum were observed following manufacturer’s instructions, and the absorbance was calculated at a wavelength of 450 nm by a microplate reader.

### Dual-luciferase reporter assay

Initially, the binding site of miR-374a-5p on WNT5A was predicted using the TargetScan website (http://www.targetscan.org/vert_71/), followed by amplifying the fragment. Next, the amplified fragment was inserted into the psiCHECK-2 vector to create a WNT5A wild-type (WT) plasmid. To construct the WNT5A mutant (MUT) plasmid, gene site mutagenesis technology was deployed to mutate some of the nucleotides in the binding site fragment. The constructed WNT5A gene dual luciferase reporter plasmid was co-transfected with either miR-374a-5p mimic or miR-374a-5p NC into 293 T cells (HG-NC071, HonorGene). Each group was subjected to luciferase activity detection, following the manufacturer’s guidelines for the Dual-Luciferase Report Gene Assay Kit (E1910, Promega).

### Ethical approval statement

The study was conducted with the approval of the ethics review committee of The Affiliated Changsha Hospital of Xiangya School of Medicine, Central South University (2022-7). All methods were performed in accordance with the relevant guidelines and regulations.

### Statistical analysis

The experiments were conducted a minimum of three times. GraphPad Prism 8.0 software was utilized for data analysis, and the results were displayed as means ± standard deviation for all quantitative data. Initially, normality and homogeneity of variance tests were conducted, revealing equal variances and normal data distribution. Unpaired t-tests were implemented for between-group comparisons, while one-way analysis of variance (ANOVA) was used for comparisons among multiple groups. Results were marked statistically significant when P < 0.05.

## Results

### miR-374a-5p was upregulated in IA patients and affected VSMC phenotypic transformation

**Figure 1:**
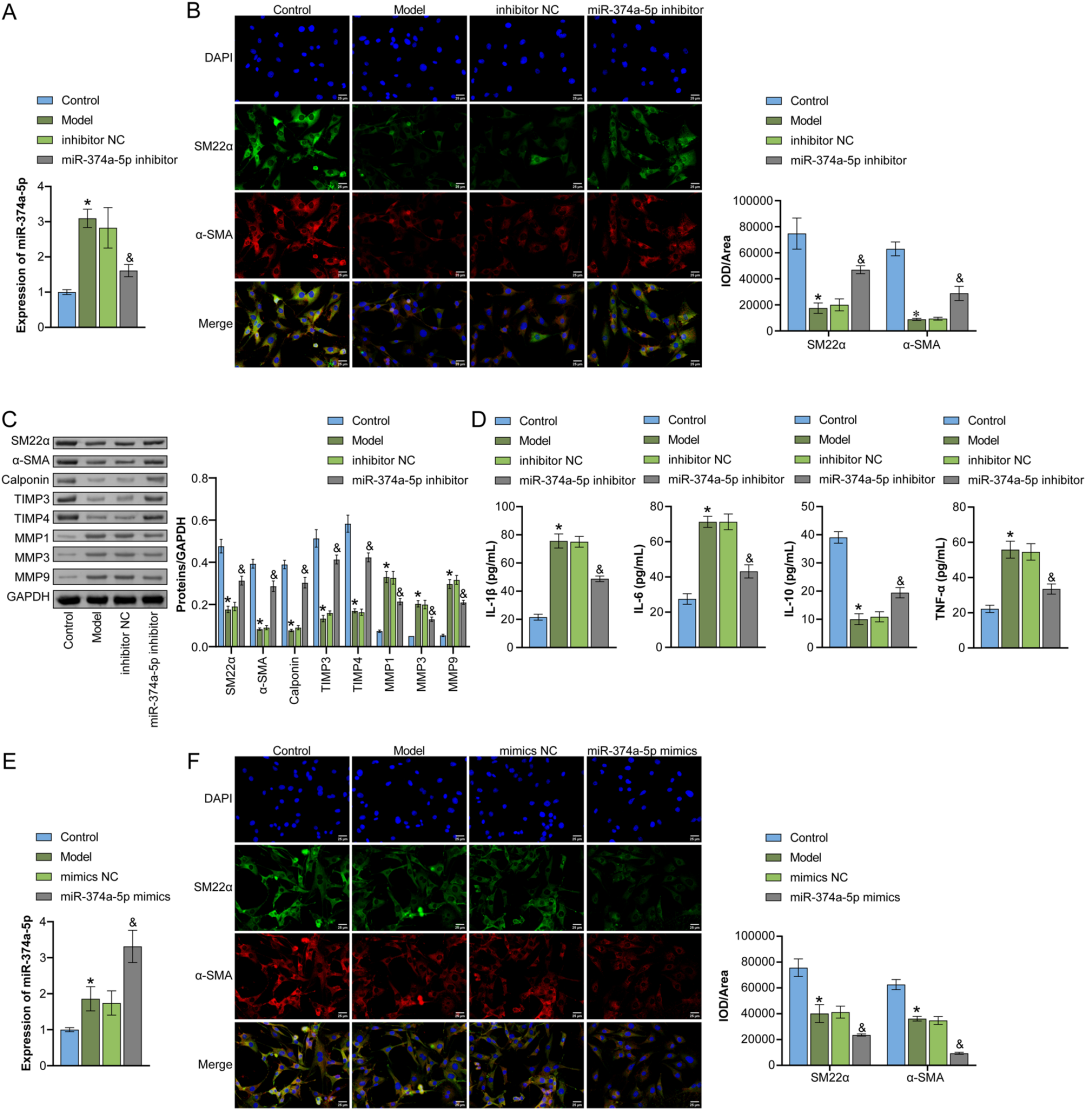
miR-374a-5p regulated VSMC phenotypic transformation (**A**) qRT-PCR analysis of miR-374a-5p expression. (**B**) IF double-staining detection of contractile markers (SM22α and α-SMA) expression in VSMCs. Scale bar = 25 μm. (**C**) WB analysis of SM22α, α-SMA, Calponin, MMP1, MMP3, MMP9, TIMP3, and TIMP4 expression. (**D**) ELISA measurement of TNF-α, IL-1β, IL-6, and IL-10 levels. *P < 0.05 vs. Control, &P < 0.05 vs. inhibitor NC. (E) qRT-PCR analysis of miR-374a-5p expression. (**F**) IF double-staining detection of SM22α and α-SMA expression in VSMCs. Scale bar = 25 μm. *P < 0.05 vs. Control, ^&^P < 0.05 vs. mimics NC. n = 3.

Firstly, we detected the expression of miR-374a-5p, SM22α, α-SMA, CD86 and CD206 in human IA and normal tissues. Compared with the Normal group, the levels of miR-374a-5p and CD86 in the IA group were significantly increased, and SM22α, α-SMA and CD206 were significantly decreased (Fig. S2A). To further evaluate the cell distribution of miR-374a-5b in the arterial wall, we performed FISH-IF with the macrophage marker CD68 and VSMC marker SM22-α, respectively. As shown in Fig. S2B, miR-374a-5b (red) and CD68/SM22-α (green) were co-localized in the arteries. Compared with the Normal group, the proportion of miR-374a-5p + SM22-α + positive cells was relatively high. Pearson correlation analysis showed that the expression of miR-374a-5p was negatively correlated with the expression of SM22α, α-SMA, and CD206, and positively correlated with the expression of CD86 (Fig. S2C). To examine the role of miR-374a-5p in VSMC phenotypic transformation, we employed a PDGF-BB-induced VSMC model, specifically to gauge the impact of miR-374a-5p on the phenomenon. The upregulation of miR-374a-5p expression in PDGF-BB-induced VSMCs was successfully suppressed using miR-374a-5p inhibitor, as depicted in Fig. 1A. Utilizing IF double-staining to observe the expression of a contractile marker in VSMC cells, a decline in SM22α and α-SMA expression in the Model group was detected against the Control group, but an increase in the miR-374a-5p inhibitor group compared to the Model group (Fig. 1B). Moreover, WB outcomes demonstrated a reduction in SM22α, α-SMA, Calponin, TIMP3, and TIMP4 expressions induced by PDGF-BB treatment, while MMP1, MMP3, and MMP9 expressions increased. Nonetheless, miR-374a-5p inhibitor reversed these protein levels, proven by the enhanced expression (Fig. 1C). In cell supernatants, ELISA analysis highlighted higher levels of IL-1β, IL-6, and TNF-α and decreased IL-10 levels in the Model group relative to the Control group. However, the miR-374a-5p inhibitor group displayed the opposite trend against the Model group (Fig. 1D). In contrast, miR-374a-5p mimics reversed the increased expression of miR-374a-5p and the decreased expression of SM22α and α-SMA in PDGF-BB-induced VSMCs (Fig. 1E,F). These results indicated that miR-374a-5p was upregulated in IA patients and promoted PDGF-BB-induced VSMC phenotypic transformation.

### In vivo validation of miR-374a-5p affecting vascular remodeling in IA rats

**Figure 2:**
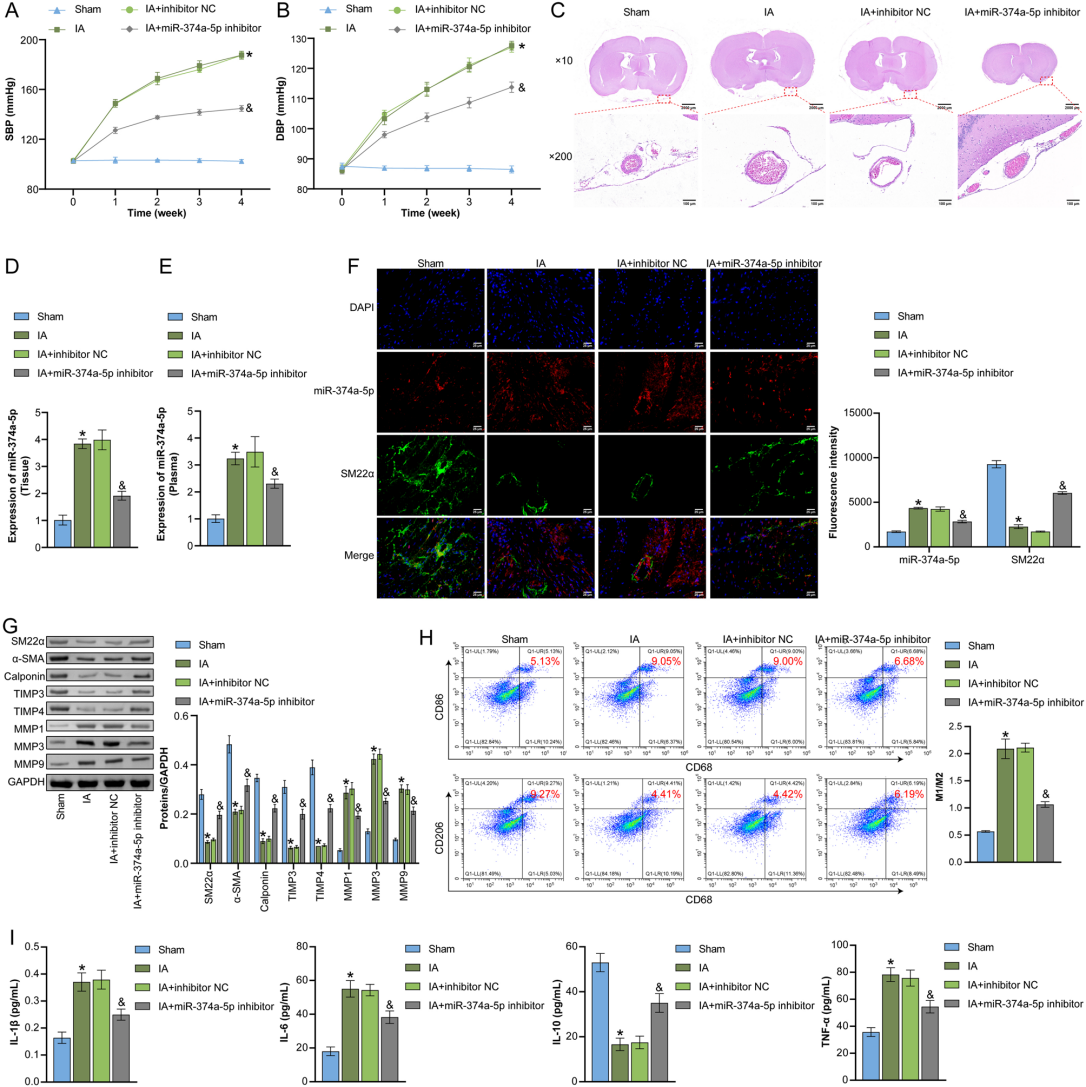
In vivo validation of miR-374a-5p affecting vascular remodeling in IA rats (**A**) and (**B**) SBP and DBP in each treatment group of mice at weeks 0, 1, 2, 3, and 4 were recorded. (**C**) HE staining to observe vascular pathological morphology. Scale bar = 100 μm and 2000 μm. (**D**) and (**E**) qRT-PCR analysis of miR-374a-5p expression in vascular wall tissue and plasma of IA rats. (**F**) The co-localization and expression of miR-374a-5b and SM22α in arterial wall were detected by FISH-IF. Scale bar = 25 μm. (**G**) WB analysis of SM22α, α-SMA, Calponin, MMP1, MMP3, MMP9, TIMP3, and TIMP4 expression. (**H**) Flow cytometry to detect infiltration of M1 macrophages (CD86) and M2 macrophages (CD206). (**I**) ELISA measurement of TNF-α, IL-1β, IL-6, and IL-10 levels. *P < 0.05 vs. Sham, ^&^P < 0.05 vs. IA + inhibitor NC. n = 3.

Next, we utilized an elastase-induced IA rat model to further investigate the effect of miR-374a-5p on vascular remodeling in IA rats. As shown in Fig. 2A,B, IA rats exhibited a significant increase in SBP and DBP, which was partially alleviated upon transfected with miR-374a-5p inhibitor. HE staining showed that the IA group had a disrupted endothelial layer, decreased VSMC count and layer number, and inflammatory cell infiltration compared to the Sham group. Still, these symptoms were improved in the IA + miR-374a-5p inhibitor group (Fig. 2C and Fig. S3). qRT-PCR results indicated the expression of miR-374a-5p was successfully suppressed in vascular wall tissue and plasma of IA rats (Fig. 2D,E). FISH-IF results showed that miR-374a-5b and SM22-α were co-localized in aneurysm vessels. The expression of miR-374a-5p was up-regulated in IA rats, which was significantly decreased after the application of miR-374a-5p inhibitor (Fig. 2F). In addition, compared to the Sham group, expression levels of SM22α, α-SMA, Calponin, TIMP3, and TIMP4 decreased, while MMP1, MMP3, and MMP9 increased in the serum of IA rats. However, miR-374a-5p inhibitor reversed these indicators (Fig. 2G). Moreover, the IA group had a higher M1/M2 ratio than the Sham group, while the IA + miR-374a-5p inhibitor group showed opposite trends to those observed in the IA group (Fig. 2H). Figure 2I illustrated that, in IA rats, serum levels of IL-1β, IL-6, and TNF-α were observed to rise significantly in comparison to the Sham group. On the other hand, the levels of IL-10 dropped correspondingly. However, by suppressing miR-374a-5p, these changes were reversed. The above data suggested that miR-374a-5p influenced vascular remodeling in IA rats.

### miR-374a-5p targeted WNT signaling

**Figure 3:**
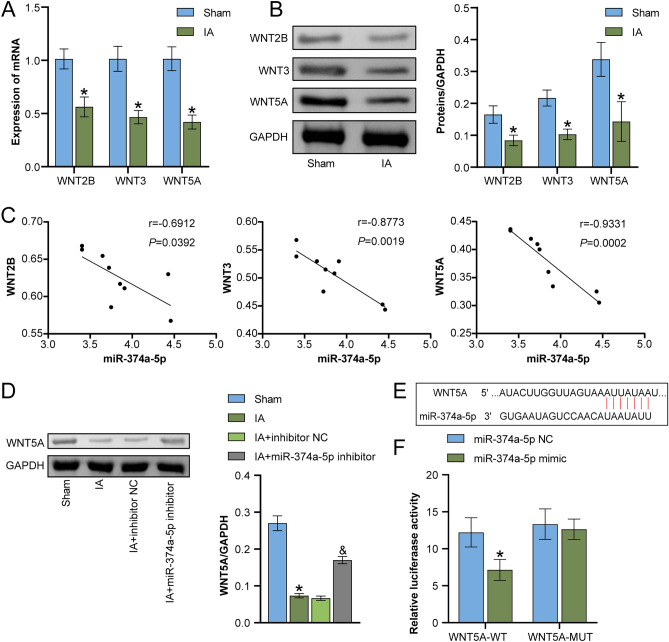
miR-374a-5p targeted WNT signaling (**A**) qRT-PCR detection of WNT5A, WNT3, and WNT2B expression. (**B**) WB detection of WNT5A, WNT3, and WNT2B expression. *P < 0.05 vs. Sham. (**C**) Pearson correlation analysis of the expression of miR-374a-5p and WNT5A, WNT3, and WNT2B. (**D**) WB detection of WNT5A expression. *P < 0.05 vs. Sham, ^&^P < 0.05 vs. IA + inhibitor NC. (**E**) Bioinformatics prediction of miR-374a-5p targeting of WNT signaling (WNT2B, WNT3, and WNT5A). (**F**) To validate the targeting relationship between miR-374a-5p and WNTA5, a dual luciferase reporter assay was conducted. *P < 0.05 vs. miR-374a-5p NC. n = 3.

Using the TargetScan database, we anticipated the potential targeting impact of miR-374a-5p on the WNT signaling pathway. Subsequently, qRT-PCR and WB analysis revealed that the mRNA and protein expression levels of WNT2B, WNT3, and WNT5A happened to be markedly lower in IA rats when compared to the Sham group (Fig. 3A,B). Pearson analysis of the correlation between miR-374a-5p and WNT signaling (WNT2B, WNT3, and WNT5A) in IA rats was shown in Fig. 3C, and the correlation with WNT5A was the greatest, thus WNT5A was selected for further study. We also found that miR-374a-5p inhibitor upregulated the expression of WNT5A in IA rats (Fig. 3D). As Fig. 3E illustrated, the bioinformatics predicted that miR-374a-5p targeted WNT5A. Further reinforced by dual-luciferase reporter assays, it was discovered that miR-374a-5p mimics reduced WT-WNT5A luciferase activity when compared to miR-128-3p NC, while MUT-WNT5A had no significant difference. This strengthened the targeting relationship between miR-374a-5p and WNT5A (Fig. 3F). Overall, these results signified that WNT5A signaling was targeted by miR-374a-5p.

### WNT5A-mediated miR-374a-5p affected VSMC phenotypic transformation

**Figure 4:**
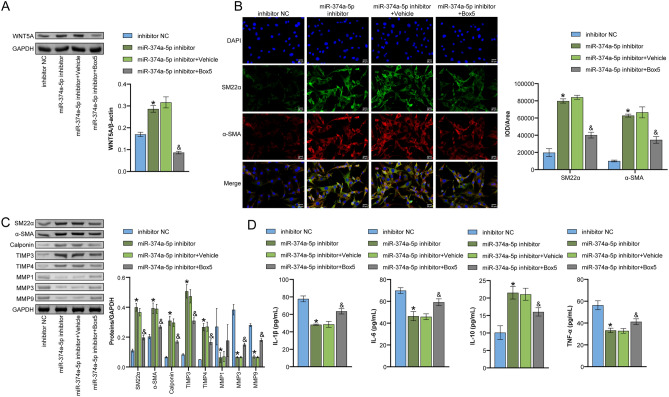
WNT5A-mediated miR-374a-5p affected VSMC phenotypic transformation (**A**) WB detection of intracellular WNT5A signal expression. (**B**) IF double-staining to detect the expression of smooth muscle cell contraction markers (SM22α and α-SMA). Scale bar = 25 μm. (**C**) WB detection of SM22α, α-SMA, Calponin, MMP1, MMP3, MMP9, TIMP3, and TIMP4 expression. (**D**) ELISA measurement of TNF-α, IL-1β, IL-6, and IL-10 levels. *P < 0.05 vs. inhibitor NC. ^&^P < 0.05 vs. miR-374a-5p inhibitor + Vehicle. n = 3.

To investigate whether miR-374a-5p affects VSMC phenotypic transformation through WNT5A, we performed Box5 intervention on the cells. First, the expression of WNT5A was detected by WB. Significantly, we noticed that relative to the inhibitor NC group, WNT5A expression exhibited a significant increase in the miR-374a-5p inhibitor group. In contrast, WNT5A expression was reduced following co-treatment with Box5, as per the data displayed in Fig. 4A. Further, the IF analysis showed that miR-374a-5p inhibitor promoted SM22α and α-SMA expression, and partially rescued the effect of miR-374a-5p when co-treated with Box5 (Fig. 4B). The WB and ELISA findings indicated that Box5 treatment led to a reversal concerning the upregulation of SM22α, α-SMA, Calponin, TIMP3, and TIMP4 expression, and IL-10 levels. It also led to the downregulation of MMP1, MMP3, and MMP9 expression, and IL-1β, IL-6, and TNF-α levels induced by miR-374a-5p inhibitor (Fig. 4C,D). These outcomes suggested a mediation of WNT5A for the effects induced by miR-374a-5p on VSMC phenotypic transformation.

### miR-374a-5p affected M1 macrophage polarization through WNT5A signaling

**Figure 5:**
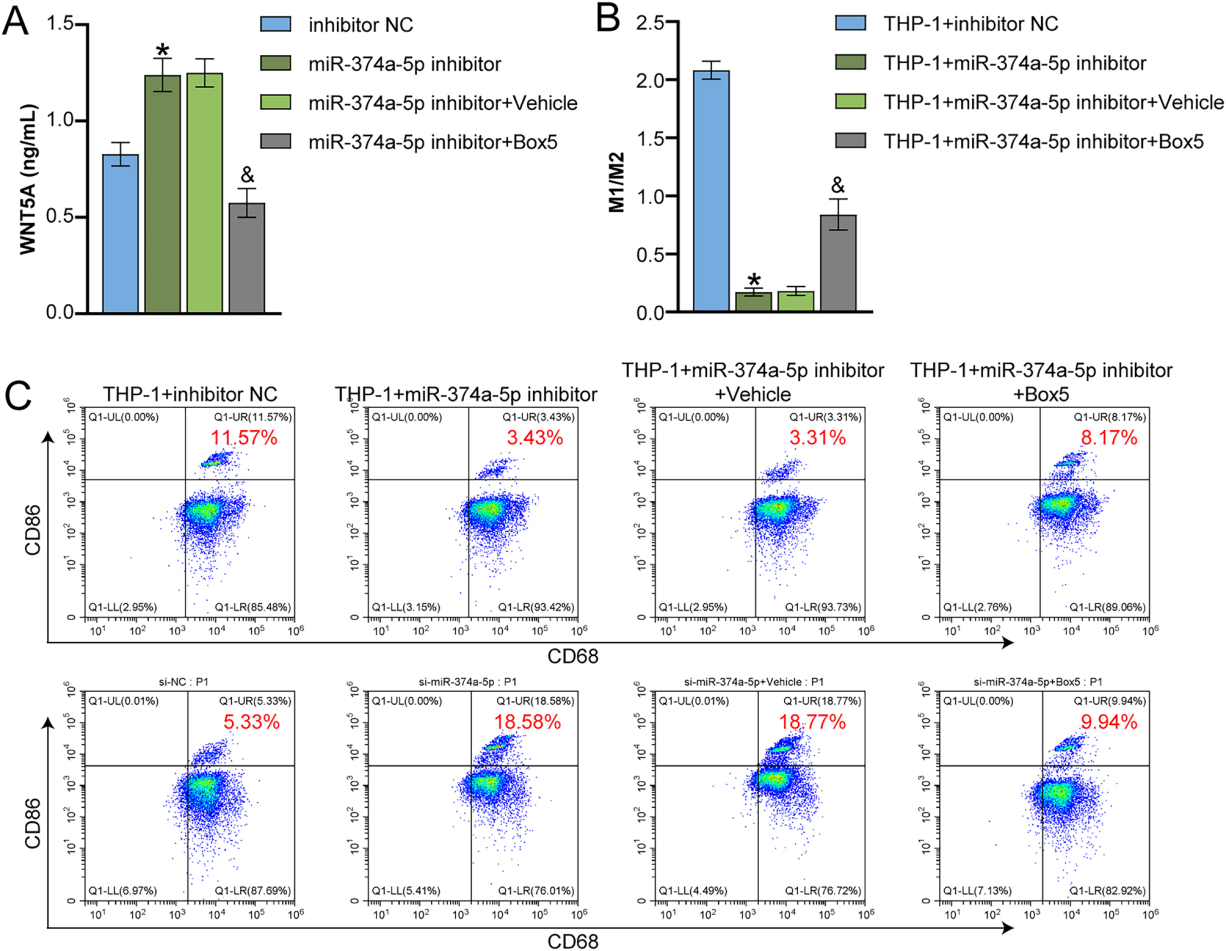
miR-374a-5p affected M1 macrophage polarization through WNT5A signaling (**A**) ELISA measurement of WNT5A level in the cell supernatant. (**B** and **C**) Flow cytometry analysis of the ratio of M1 macrophages (CD86) and M2 macrophages (CD206). *P < 0.05 vs. inhibitor NC. ^&^P < 0.05 vs. miR-374a-5p inhibitor + Vehicle. n = 3.

Finally, we investigated the effects of WNT5A on THP-1 cell-derived macrophage polarization. We first measured the level of WNT5A in VSMCs using ELISA. The data in Fig. 5A indicated that WNT5A expression levels decreased significantly following treatment with Box5 compared to the miR-374a-5p inhibitor group, where they exhibited a rise. Next, THP-1 cells were co-cultured with PDGF-BB-treated VSMC cells, and flow cytometry analysis showed that the M1/M2 ratio was significantly decreased in the THP-1 + miR-374a-5p inhibitor group compared to the inhibitor NC group, and Box5 reversed the effects of miR-374a-5p inhibitor (Fig. 5B,C). These results suggested that miR-374a-5p affected M1 macrophage polarization through the WNT5A signaling.

## Discussion

Given the detrimental consequences of subarachnoid hemorrhage due to IA rupture, preventative interventions to impede lesion rupture are essential for public health. Previous studies have shown an increased expression of miR-374a-5p in IA, yet its specific role and mechanisms remain elusive. In our study, miR-374a-5p was upregulated in IA patients. Importantly, miR-374a-5p was found to regulate VSMC phenotypic transformation and M1 macrophage polarization by targeting WNT5A, thereby impacting the progression of IA. Our findings contributed novel insight into the specific mechanism by which miR-374a-5p impacts the progression of IA via WNT5A signaling, signifying the innovative nature of our study.

VSMCs are highly specialized contractile cells with considerable plasticity, displaying marked phenotype changes upon stimulation^25,26^. It’s worth noting that VSMC phenotypic transformation in IA pathogenesis plays a crucial role in IA formation, development, and rupture^27^. Additionally, Macrophages are key effector cells in the progression of IA^28^. In IA development, there is increased polarization towards the M1 phenotype, and limiting macrophage M1 polarization may prevent IA formation and rupture^5,29^. Specifically, inflammatory cytokines secreted by M1 cells trigger pathological changes in the IA wall, such as TNF-α, which, on the one hand, initiates VSMC phenotypic transformation from contractile to pro-inflammatory and matrix remodeling phenotypes. On the other hand, stimulated VSMCs propagate the inflammatory cascade by secreting cytokines^30^, ultimately driving macrophage M1 polarization. In summary, the interaction between M1 macrophages and VSMCs may exacerbate the progression of IA through a positive feedback loop. Our study has revealed that SM22α, α-SMA, Calponin, TIMP3, TIMP4, and IL-10 levels were reduced in PDGF-BB-induced VSMCs, while MMP1, MMP3, MMP9, IL-1β, IL-6, and TNF-α levels were elevated. Notably, a similar phenomenon was observed in IA rats. Furthermore, IA rats exhibited a higher M1/M2 ratio, SBP, and DBP than normal rats. Additionally, IA rats presented with significant vascular lesions. These findings suggested that VSMC phenotypic transformation and macrophage polarization play important roles in the occurrence and development of IA.

Presently, many studies are focusing on the impact of miRNAs in the creation and progression of IA. There is evidence to suggest that miRNAs contribute to the regulation of VSMC proliferation, migration, and apoptosis. For instance, Hou et al. reported the inhibitory effect of miRNA-370-3p on VSMC proliferation by targeting the KDR/AKT signal pathway and its role in IA occurrence and development^31^. In a similar vein, abnormal expression of miR-29b and miRNA-9 promotes the occurrence and rupture of IA by mediating VSMC phenotypes and inhibiting their proliferation^32,33^. These studies provide a basis for profound insights into pathology, and the exploration of potential clinical applications that could facilitate the prevention and treatment of IA. Recent studies have shown that miR-374a-5p is highly upregulated in the plasma of IA patients^12^. However, the specific mechanism underlying the role of miR-374a-5p in IA has not been fully illuminated yet and requires further investigation. Our study further confirmed that miR-374a-5p was overexpressed in the arteries of IA patients, mainly expressed in VSMCs. In vitro*,* knocking down miR-374a-5p reversed the promotion of SM22α and α-SMA expression by PDGF-BB, while overexpression of miR-374a-5p has the opposite effect. Meanwhile, downregulating miR-374a-5p reversed the decrease in Calponin, TIMP3, TIMP4, and IL-10 levels caused by PDGF-BB, and further reduced the levels of MMP1, MMP3, MMP9, IL-1β, IL-6, and TNF-α. These data were further validated in vivo, suggesting that miR-374a-5p regulated PDGF-BB-induced phenotypic transformation of VSMCs. Furthermore, in animal experiments, IA rats exhibited a significant increase in SBP and DBP, accompanied by vascular lesions, which was alleviated by miR-374a-5p downregulation. These findings indicated that miR-374a-5p impacted vascular remodeling in IA rats.

WNT5A is a representative WNT ligand that regulates cellular functions via the non-canonical WNT5A pathway^34^. Prior studies have indicated that WNT5A may participate in the pathogenesis of vascular disease by regulating the behavior of macrophages and VSMCs^16^. However, little is currently understood regarding the role of WNT5A signaling in the pathophysiology of IA. In this research, combining bioinformatics analysis and experimental validations, we found that miR-374a-5p targeted and negatively regulated WNT5A. miR-374a-5p inhibitor led to an increase in the expression of SM22α, α-SMA, Calponin, TIMP3, and TIMP4 as well as IL-10 content, and a decrease in the expression of MMP1, MMP3, and MMP9 and the content of IL-1β, IL-6, and TNF-α. The co-administration of Box5 reversed the effects of miR-374a-5p inhibitor. These results suggested that WNT5A mediated the effects of miR-374a-5p on the phenotypic transformation of VSMCs. Additionally, miR-374a-5p inhibitor led to a significantly decrease in M1/M2 ratio, which was reversed by Box5 the effects of. The above results indicated that miR-374a-5p affected M1 macrophage polarization via the WNT5A signaling.

In this study, we discovered that downregulating miR-374a-5p promoted VSMC phenotypic transformation to the contractile phenotype and inhibited M1 macrophage polarization, an effect reversed by the WNT5A antagonist Box5. Nevertheless, our research still has many limitations. Firstly, the clinical sample size of this study is limited, and larger sample sizes can be used for future verification. Secondly, further in-depth studies are needed to explore the mechanism of how PEGF-BB and IA models induce miR-374a-5p overexpression. Additionally, future research can further examine the expression of miR-374a-5p in other cell types, such as aortic endothelial cells, to reveal its role and regulatory mechanisms in different cell types. Furthermore, additional studies and clinical trials are needed to determine whether reducing BP can reduce the severity or mortality of IA. Overall, our results provide new evidence that miR-374a-5p regulated VSMC phenotypic transformation and M1 macrophage polarization via WNT5A, thus affecting the progression of IA. These results provide new insights into the development of new drugs for the treatment of IA, with the miR-374a-5p/WNT5A axis offering promise as a potential target for IA treatment, providing a fresh direction for future drug research and development.

### Supplementary Information

Supplementary Figures.

## Data Availability

The data analyzed for the study are included in the manuscript. Raw data can be obtained from the first or corresponding author upon reasonable request.
