# Supplementary material for: WNTA5-mediated miR-374a-5p regulates vascular smooth muscle cell phenotype transformation and M1 macrophage polarization impacting intracranial aneurysm progression
Source: Sci Rep. 2024 Jan 4;14:559. doi: 10.1038/s41598-024-51243-z (PMC10766994; doi:10.1038/s41598-024-51243-z)
Supplement: Supplementary file 1 — Supplementary Figures. [file 41598_2024_51243_MOESM1_ESM.docx]

## Supplementary Materials


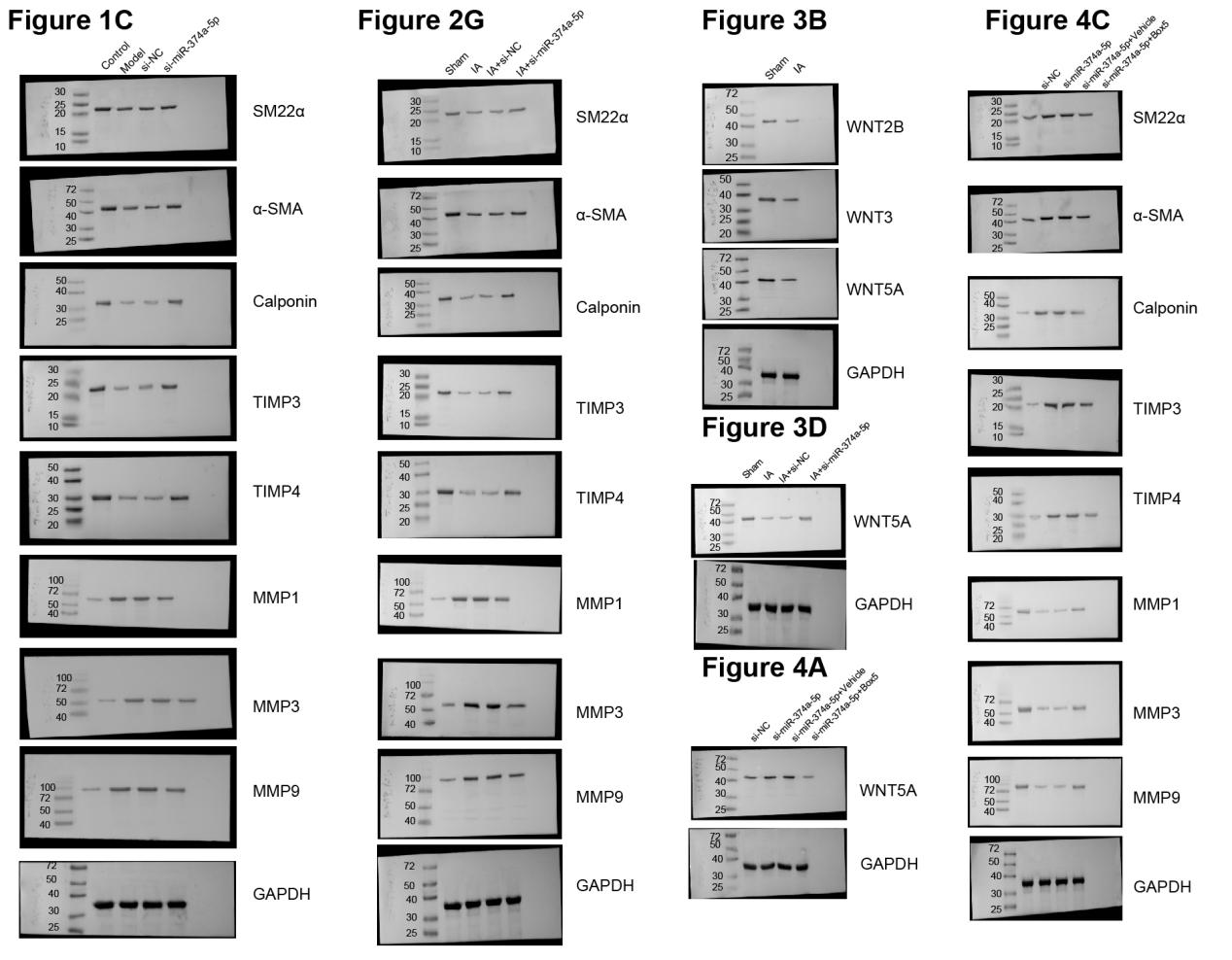


**Figure S1.** Original images of uncropped WB bands.


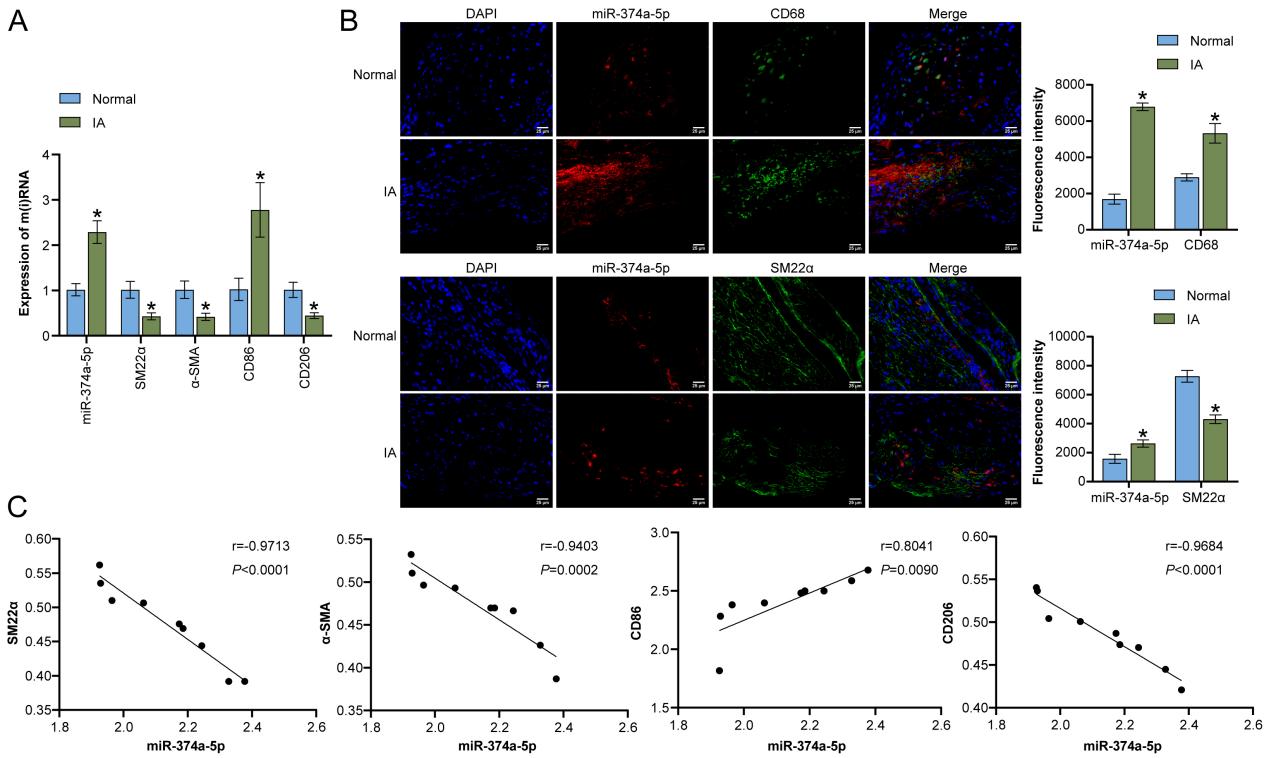


**Figure S2 miR-374a-5p was upregulated in IA patients** (A) qRT-PCR analysis of miR-374a-5p expression in Normal and IA tissues. (B) FISH-IF was used to evaluate the cellular distribution of miR-374a-5b in the arterial wall. (C) Pearson correlation analysis of miR-374a-5p and SM22α, α-SMA, CD86 and CD206. *P<0.05 vs. Normal. Normal (n=4), IA (n=2).


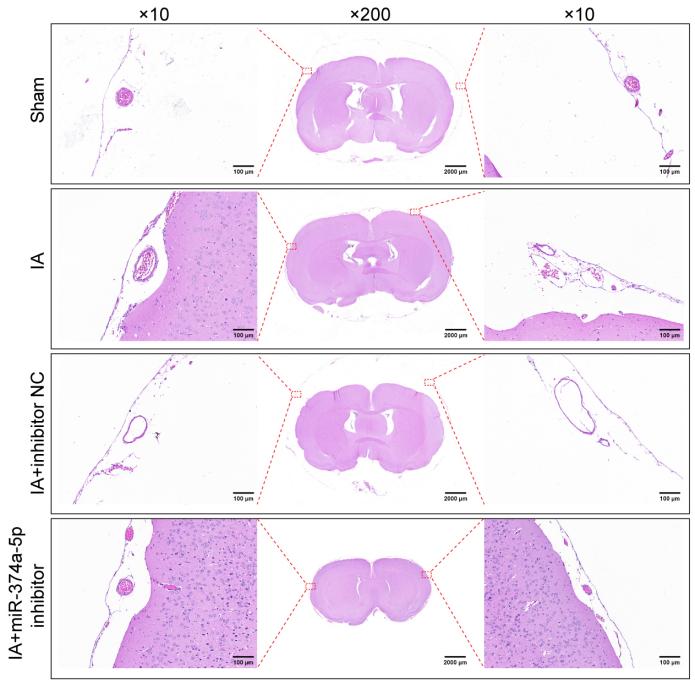


**Figure S3 HE staining to observe vascular pathological morphology.** **Scale bar=100 μm and 2000 μm. n=3.**
